# Young Age and Concomitant Cannabis (THC) and Ethanol (EtOH) Exposure Enhances Rat Brain Damage Through Decreased Cerebral Mitochondrial Respiration

**DOI:** 10.3390/molecules30040918

**Published:** 2025-02-17

**Authors:** Véronique Quenardelle, Anne-Laure Charles, Anne Charloux, Jean-Sébastien Raul, Valérie Wolff, Bernard Geny

**Affiliations:** 1 Biomedicine Research Center of Strasbourg (CRBS), UR 3072, “Mitochondria, Oxidative Stress and Muscle Plasticity”, Faculty of Medicine, University of Strasbourg, 67000 Strasbourg, France; veronique.quenardelle@chru-strasbourg.fr (V.Q.); anne.laure.charles@unistra.fr (A.-L.C.); anne.charloux@chru-strasbourg.fr (A.C.); valerie.wolff@chru-strasbourg.fr (V.W.); 2 Neuro-Vascular Department, University Hospital of Strasbourg, 67091 Strasbourg, France; 3 Department of Physiology and Functional Explorations, University Hospital of Strasbourg, 67091 Strasbourg, France; 4 Toxicology Laboratory, Institute of Legal Medicine, Faculty of Medicine, University of Strasbourg, 67000 Strasbourg, France; jean-sebastien.raul@chru-strasbourg.fr

**Keywords:** cannabis, THC, ethanol, EtOH, brain, mitochondria, stroke, mitochondrial respiration, oxidative stress, hydrogen peroxide (H_2_O_2_)

## Abstract

The reason why young people taking concomitantly cannabis (THC) and ethanol (EtOH) are more prone to stroke is underresearched. To investigate whether an underlying mechanism of increased brain damage could be an impaired mitochondrial function, this experiment determined the acute effects of EtOH, both alone and associated with THC, on mitochondrial respiration and oxidative stress (hydrogen peroxide H_2_O_2_) on young (11 weeks) and middle-aged (45 weeks) brain in rats, using a high-resolution oxygraph (Oxygraph-2K, Oroboros instruments). In young brains, EtOH decreased mitochondrial respiration by −51.76 ± 2.60% (from 32.76 ± 3.82 to 17.41 ± 1.42 pmol/s/mL, *p* < 0.0001). In 45-week-old brains, the decrease was lesser, but still significant −36.0 ± 2.80% (from 30.73 ± 7.72 to 20.59 ± 5.48 pmol/s/mL, *p* < 0.0001). Concomitant THC aggravated brain mitochondrial respiration decreases at 11 weeks (−86.86 ± 1.74%, *p* < 0.0001) and at 45 weeks (−73.95 ± 3.69%, *p* < 0.0001). Such additional injury was enhanced in young brains (*p* < 0.01). H_2_O_2_ production was similar in both age groups (1.0 ± 0.2 versus 1.1 ± 0.08 pmol O_2_/s/mL) and was not modified by THC addition. In conclusion, EtOH alone significantly impairs brain mitochondrial respiration and concomitant THC further aggravates such damage, particularly in young brains. These data support the hypothesis that enhanced mitochondrial dysfunction might participate in the increased occurrence of stroke in the young and urge for better prevention against EtOH and THC addictions in adolescents.

## 1. Introduction

Cannabis and alcohol are among the most widely used drugs in the world, including in young adults. More than hundred cannabinoids have been isolated, and cannabis from Cannabis Sativa contains cannabidiol, which is the primary non-psychoactive chemical, and Δ9-tetrahydrocannabinol (THC), which induces many psychotropic effects [1,2,3]. Besides these plant-derived phytocannabinoids, synthetic THC molecules have been developed, and their use for either recreational or therapeutic purposes spread quickly. Indeed, they are used for pain relief, to treat nausea and vomiting, and for anti-inflammatory effects [4,5,6,7,8].

THC can, however, result in several psychotropic effects in cognition, motor behavior, memory, and learning impairments. Its consumption is related to addiction, leading to the occurrence of neuropsychological and psychiatric disorders. The cognitive effects may depend on the age of the consumer, since it might have effects on neurodevelopment. Indeed, exposure to cannabinoids could have remote effects when used during adolescence, a crucial time of neurodevelopment characterized by key structural and functional changes leading to cognitive maturation [9,10]. Regarding brain effects, neuroimaging showed that cannabis can alter brain activity and connectivity patterns in healthy volunteers.

Additionally, cannabis cause major cardiovascular alterations, including myocardial infarction, characterized by a worse prognosis as compared to myocardial infarction secondary to atherosclerosis [11,12,13,14,15]. Further, cannabis increases the probability of the occurrence of stroke. In particular, a temporal relationship between the use of marijuana (natural or synthetic) and stroke in young people has been described [16,17,18,19,20,21].

The pathophysiology of stroke occurring during cannabis use is still under debate. Particularly, the mechanisms underlying the increased occurrence of stroke in the young are not fully understood. In this view, the activation of the sympathetic nervous system and coagulation, together with arterial stenosis, likely play a role through ischemia–reperfusion-related necrosis. Indeed, cannabis has been involved in intracranial arterial stenosis in ischemic stroke in young patients [22], and cannabis-induced reversible cerebral vasoconstriction occur in about 1/3 of stroke cases [23].

Mechanisms that could participate in the cannabis-related deleterious effects may be found at the subcellular level. Interestingly, THC has been shown to decrease the mitochondrial respiration in several organs such as the liver [24], striated muscles, and the brain [25,26,27,28]. Furthermore, THC targets the cannabinoid receptor type I, which is in mitochondria, among various cerebral structures. Therefore, THC can modulate oxidative phosphorylation activity and may reduce mitochondrial respiration [29]. Importantly, the THC-related brain mitochondrial respiration decrease resulted in learning/memory deficits in rats [30]. In humans, evidence also supports the negative effects of cannabis use on cognition and cognitive development [31,32].

Similarly, besides deleterious effects on the liver, nerves, and striated muscles, alcohol acutely depresses the central neural activity, leading to neuropsychological and psychiatric disorders [6]. Ethanol (EtOH) is also well known to impair the mitochondrial functions in liver, heart and skeletal muscles, and in the brain [33,34,35,36]. Particularly with regard to the brain, EtOH impaired mitochondrial respiration, that, in turn, leads to neuronal death [37,38,39,40].

Since young people frequently consume both THC and EtOH concomitantly, the question of whether one drug might enhance the deleterious effect of the other is raised. For instance, such effects were shown in several studies where THC, together with EtOH, resulted in greater cardiovascular and respiratory diseases compared to each drug when used alone [41,42]. Furthermore, the deleterious cumulative effects of THC and EtOH were recently reported on mitochondrial respiration in cardiac and skeletal muscles [27,43]. Concerning the brain, accordingly, cognitive impairments were major when THC and EtOH were used simultaneously [44], but the mechanisms behind this cumulative effects have not been investigated yet.

Since the cannabis effects in stroke was shown especially in young patients, the aim of this study was to investigate whether a concomitant exposure to THC and EtOH could alter brain mitochondrial respiration, and whether such change could be enhanced by young age. We, therefore, determined the acute effects of EtOH, both alone and associated with THC, on mitochondrial respiration and oxidative stress in the brain of 11- and 45-week-old rats.

## 2. Results

### 2.1. Baseline Brain Mitochondrial Respiration and H_2_O_2_ Production

**Figure 1:**
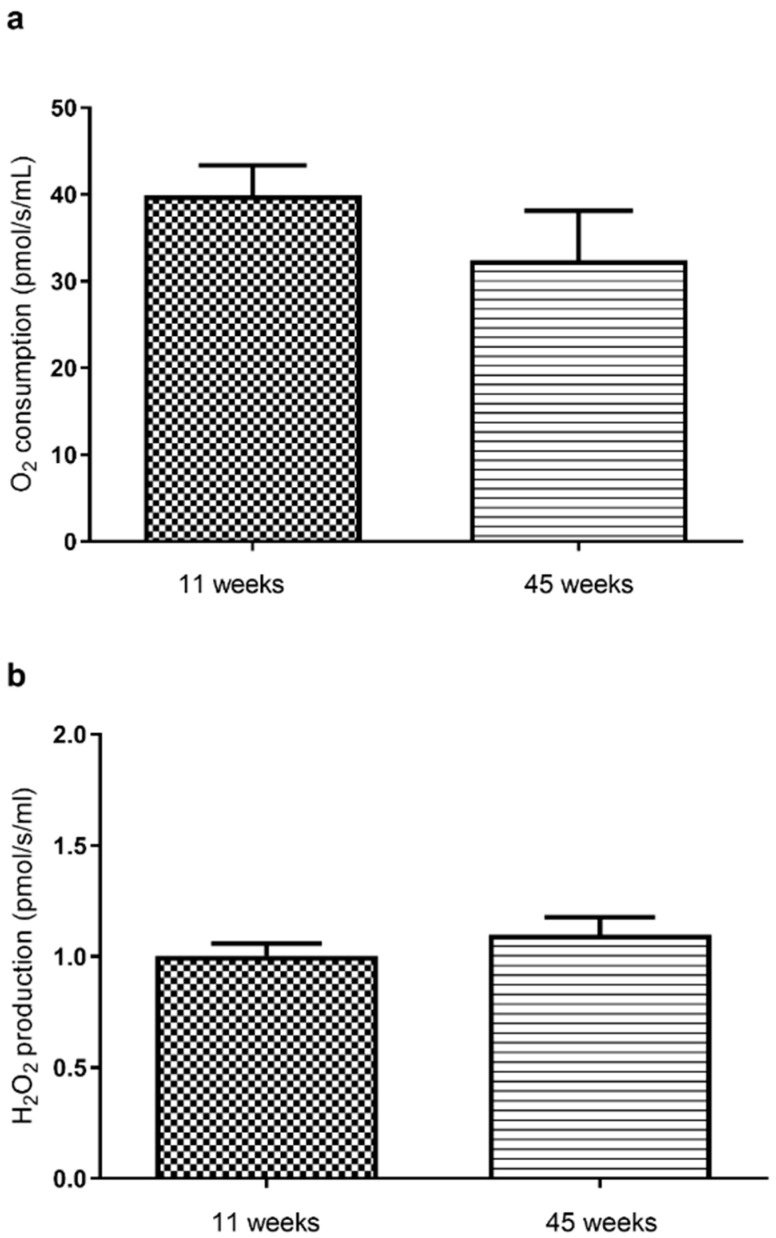
Baseline brain mitochondrial respiration (**a**) and H_2_O_2_ production (**b**) in 11- and 45-week-old rats. Baseline brain mitochondrial respiration and H_2_O_2_ production are not significantly different in 11- and 45-week-old brains, before THC or EtOH addition. Oxygen consumption was measured using a high-resolution oxygraph. OXPHOS CI was measured by injection of glutamate, malate, and ADP. Values are means ± SEM. n = 5 per group. A Mann–Whitney test was performed. H_2_O_2:_ hydrogen peroxide.

Before THC or EtOH addition, baseline mitochondrial respiration tended to decrease in 45-week-old rats, as compared to the 11-week-old rats (32.4 ± 5.7 versus 39.9 ± 3.4 pmol O_2_/s/mL, Figure 1a).

Considering H_2_O_2_ mitochondrial production, we observed no significant difference between both ages (1.0 ± 0.2 versus 1.1 ± 0.08 pmol O_2_/s/mL, in 11- and 45-week-old rat brains, respectively, Figure 1b).

### 2.2. EtOH Effects on Brain Mitochondrial Respiration and H_2_O_2_ Production

**Figure 2:**
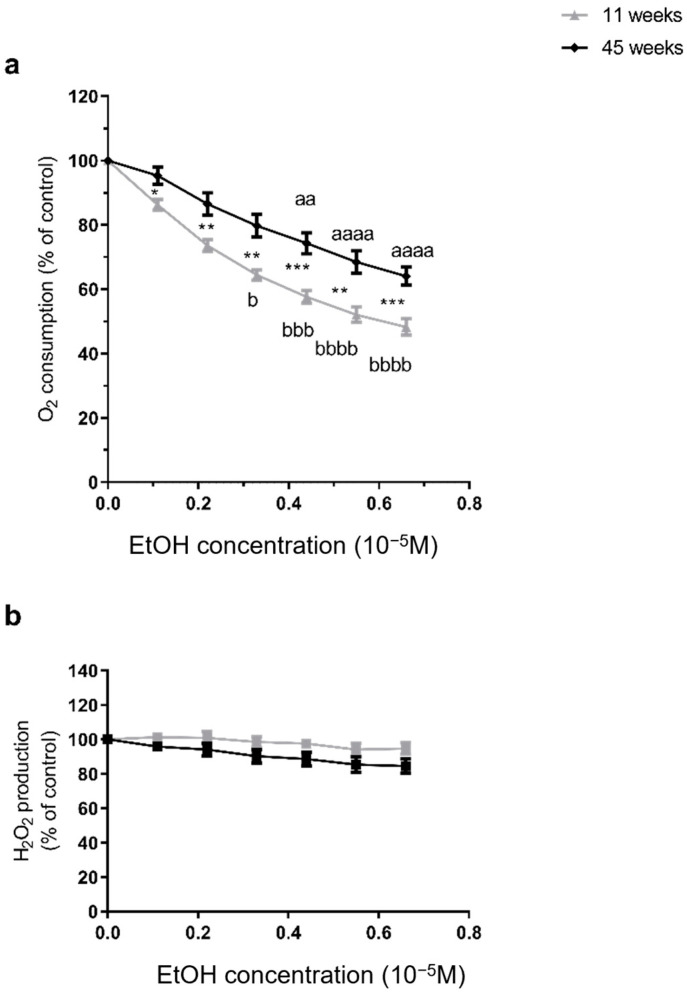
EtOH alone decreased mitochondrial respiration more severely in 11- as compared to 45-week-old rats. (**a**) Effect on mitochondrial respiration, (**b**) Effect on H_2_O_2_ production. High resolution oxygraphy was used to measure both variables. 100% corresponds to the OXPHOS CI state before any injection of EtOH. Values are means ± SEM. n = 5 per group. ANOVA was performed with the Dunnett post hoc test to analyze the evolution of variables following Ethanol exposures. Effect of increasing dose in each group (a for 45 weeks old, and b for 11 weeks old). b *p* < 0.05, aa *p* < 0.01, bbb *p* < 0.001, aaaa or bbbb *p* < 0.0001 vs. baseline. A Mann–Whitney test was used to compare the effects of age. Comparisons between groups * *p* < 0.05, ** *p* < 0.01, *** *p* < 0.001. EtOH: ethanol. H_2_O_2;_ hydrogen peroxide.

The EtOH-induced decrease in brain mitochondrial respiration was significantly worse in young brains. Thus, at 11 weeks, EtOH induced a reduction in mitochondrial respiration by −51.76 ± 2.60% for the highest dose (from 32.76 ± 3.82 to 17.41 ± 1.42 pmol/s/mL, *p* < 0.0001). In the 45-week-old rats, the decrease was lighter but still significant at −36.0 ± 2.80% (from 30.73 ± 7.72 to 20.59 ± 5.48 pmol/s/mL, *p* < 0.0001). Accordingly, IC50 (THC needed to inhibit 50% of mitochondrial respiration) was reached at 0.58 × 10^−5^ M of EtOH, only in the younger group (Figure 2a). Furthermore, O_2_ consumption was significantly more impaired at 11 weeks than at 45 weeks, regardless of the dose (−51.76 ± 2.60 vs. −36.0 ± 2.80%, *p* < 0.001, at the maximal dose).

Concerning reactive oxygen species, H_2_O_2_ production remained unchanged after EtOH, regardless of the age. In 11-week-old rats, EtOH did not modify mitochondrial H_2_O_2_ production. Similarly, no change was observed in 45-week-old rats (Figure 2b).

### 2.3. Effects of THC Associated with EtOH on Brain Mitochondrial Respiration and H_2_O_2_ Production

**Figure 3:**
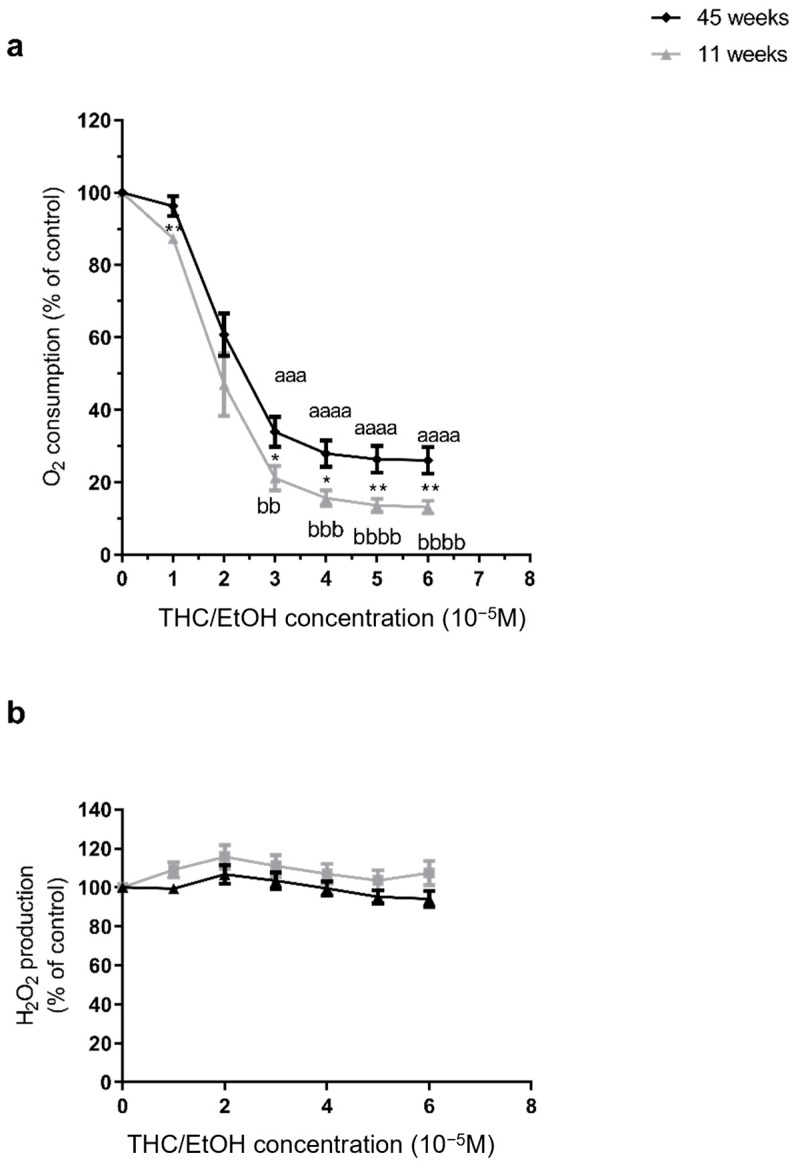
Dose–response effects of THC associated with EtOH on brain mitochondrial respiration in 11- and 45-week-old rats. (**a**) Effect on mitochondrial respiration, (**b**) Effect on H_2_O_2_ production. High resolution oxygraphy was used to measure both variables. 100% corresponds to the OXPHOS CI state before any injection of EtOH. Values are means ± SEM. n = 5 per group. ANOVA was performed with the Dunnett post hoc test to analyze the evolution of variables following Ethanol exposures. Effect of increasing dose in each group (a for 45 weeks old, and b for 11 weeks old). bb *p* < 0.01, aaa or bbb *p* < 0.001, aaaa or bbbb *p* < 0.0001 vs. baseline. A Mann–Whitney test was used to compare the effects of age. Comparisons between groups * *p* < 0.05, ** *p* < 0.01. EtOH: ethanol. THC: tetrahydrocannabinoid.

THC combined with EtOH dramatically impaired brain mitochondrial respiration. Thus, THC dissolved in EtOH significantly impaired brain mitochondrial respiration, for rats of 11 weeks of age, the loss was −86.86 ± 1.74% at 6 × 10^−5^ M, (from 36.31 ± 4.26 to 4.81 ± 0.48 pmol/s/mL, *p* < 0.0001). At 45 weeks of age, the decrease was smaller but still significant (−73.95 ± 3.69% at 6 × 10^−5^ M, from 29.73 ± 7.58 to 7.95 ± 3.24 pmol/s/mL, *p* < 0.001). Mitochondrial O_2_ consumption was significantly lower at 11 weeks than at 45 weeks regardless of the dose (−86.86 ± 1.74 vs. −73.95 ± 3.69%, *p* < 0.01, at 6 × 10^−5^ M, *p* < 0.01. Accordingly, the IC50 (THC needed to inhibit 50% of the mitochondrial respiration) was reached in both groups, but with a lower dose in young brains (2 × 10^−5^ M, and 2.44 × 10^−5^ M for 11 and 45 weeks, respectively, Figure 3a).

H_2_O_2_ production remained unchanged after THC combined with EtOH, regardless of the age at 11 weeks, THC combined with EtOH did not modify significantly ROS production (+7.37 ± 6.15% at 6 × 10^−5^ M). At 45 weeks, ROS production was not modified significantly (−5.86± 4.18% at 6 × 10^−5^ M. There was no significant difference between the two age groups (Figure 3b).

### 2.4. Concomitant THC Enhanced the Deleterious Effects of EtOH on Brain Mitochondrial Respiration in 11- and 45-Week-Old Rats

**Figure 4:**
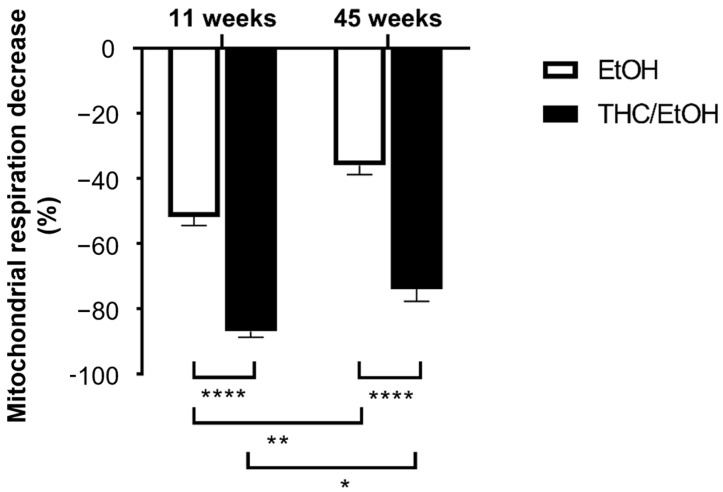
Concomitant THC and EtOH are more deleterious than EtOH alone, both in 11- and 45-week-old brain mitochondrial respiration. Values are means ± SEM., n = 5 per group. A two-way ANOVA was performed with Sidak’s post hoc test * *p* < 0.05, ** *p* < 0.01, **** *p* < 0.0001. EtOH: ethanol. THC; tetrahydrocannabinoid.

At 11 weeks, the THC combined with EtOH-induced decrease in mitochondrial respiration was significantly greater than EtOH alone (−86.9 ± 1.7 and −51.8 ± 2.6%, *p* < 0.0001, at the higher dose 6 × 10^−5^ M THC/EtOH or 0.66 × 10^−5^ M EtOH alone, respectively), as shown in Figure 4.

At 45 weeks, THC associated with EtOH also impaired the brain mitochondrial respiration to a greater degree (−73.9 ± 3.7 and −36.0 ± 2.8%, *p* < 0.0001, at the higher dose 6 × 10^−5^ M THC/EtOH or 0.66 × 10^−5^ M EtOH alone, respectively).

### 2.5. Relative Contributions of Ethanol and THC on Brain Mitochondrial Respiration Alterations

**Figure 5:**
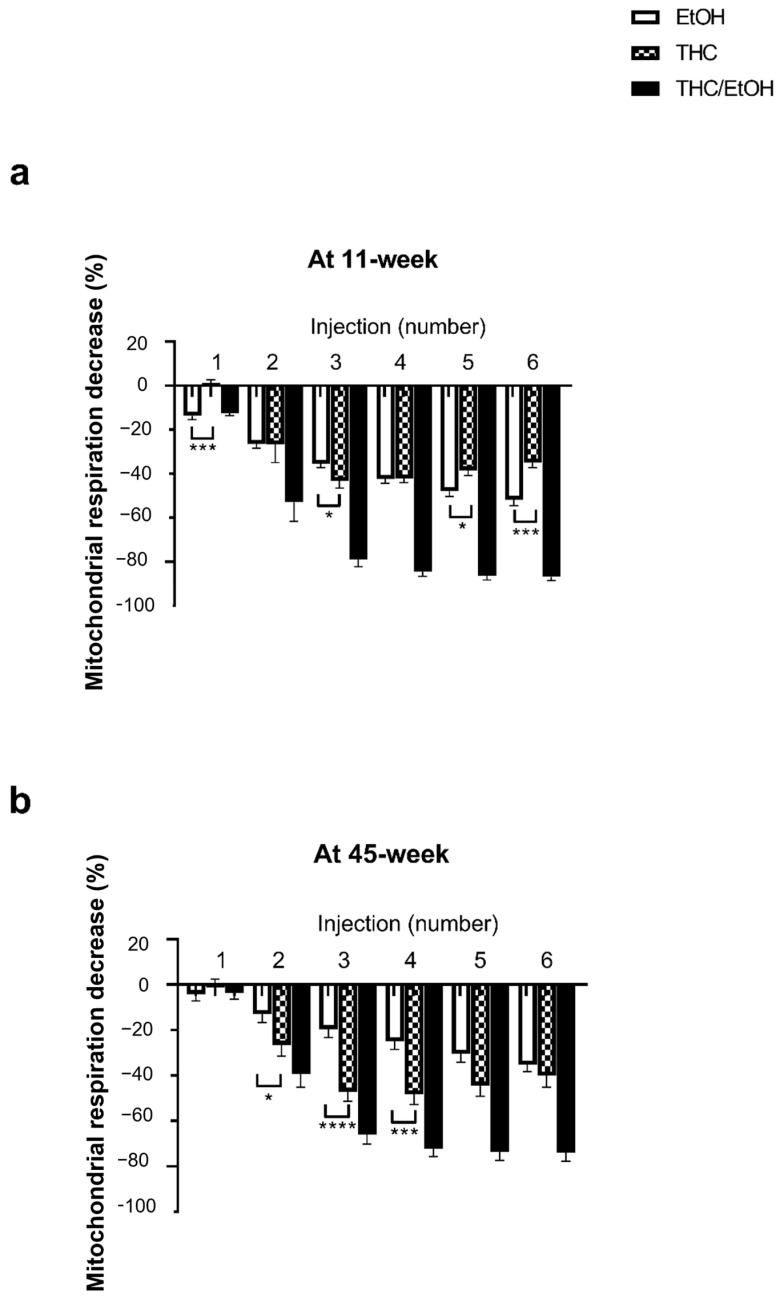
Relative contributions of ethanol and THC on brain mitochondrial respiration. Values are means ± SEM. n = 5 in each group. A Mann–Whitney test was performed * *p* < 0.05, *** *p* < 0.001, **** *p* < 0.0001 EtOH vs. THC effects. EtOH: ethanol. THC: tetrahydrocannabinoid.

To go further, as previously reported [27,43], we determined the percent (%) changes in brain mitochondrial respiration related to THC alone, by subtracting the EtOH alone effect from the global effect of THC associated with EtOH. In both ages, EtOH-induced brain alteration linearly increased with increasing EtOH concentrations. Concerning THC, the peak reduction in brain mitochondrial respiration was attained at the third dose, followed by a near plateau with higher THC doses (Figure 5).

## 3. Discussion

The main results of this study are that EtOH alone significantly decreases brain mitochondrial respiration, and that concomitant THC further aggravates such impairment. Age modulates these deleterious effects, and young brains are more prone to damage than older brains. These data improve our knowledge on the cerebral effects of both drugs, support a mechanistic involvement of mitochondrial dysfunction, and might explain the higher occurrence of THC-related stroke in young people.

### 3.1. EtOH Significantly Decreases Brain Mitochondrial Respiration

EtOH alone decreased the brain mitochondrial respiration significantly in a dose-dependent manner. This is consistent with previous reports supporting the idea that alcohol impairs brain mitochondrial functions, both after chronic and acute exposures [34]. Thus, chronic alcohol fed-mice demonstrated decreased complex activity in isolated brain mitochondria [37]. The alcohol being injected intraperitoneally also decreased mitochondrial respiration and ATP production rates [38]. Similarly, there was a significant inhibition of brain mitochondrial respiration two hours after acute intraperitoneal alcohol injection [45]. In vitro, EtOH inhibited depolarization mediated OXPHOS stimulation [46] and cerebellar cells demonstrated impaired mitochondrial function. These data are in line with our results and strongly support that mitochondrial dysfunction may contribute to brain damage.

An increase in oxidative stress was associated with the deleterious effects of alcohol-related mitochondrial function [27,28,34,47]. However, published data are controversial and Ribiere et al. observed that brain mitochondrial superoxide production was not modified after acute ethanol exposure [45]. In our experiment, and according to the latter publication, hydrogen peroxide production did not change. This might be related to the decreased mitochondrial respiration observed, and thus, to a decreased reactive oxygen species production linked to reduced complex I and III activity. On the other hand, it might also signify that superoxide anion was not fully degraded by superoxide dismutase. Future studies are needed to further investigate the ROS responses to EtOH and THC exposure in brain.

### 3.2. Concomitant THC and EtOH Further Aggravates Brain Mitochondrial Respiration Impairment

Since EtOH and THC are often used simultaneously, it was interesting to determine their combined effects on brain mitochondria. Indeed, previous data supported direct deleterious effects of THC or EtOH on several organs including the brain and the heart. Despite the fact that they are quickly absorbed and present in tissues [24,48,49,50,51,52,53,54] relatively few data are available on the potential mitochondrial interactions of these two drugs. Interestingly, our study demonstrates that THC addition significantly aggravated the deleterious effects of EtOH alone. This is in line with data obtained in other tissues. Thus, concomitant EtOH and THC aggravated the effects of each drug alone on cardiac and skeletal muscle’s mitochondrial respiration [27,43]. This has clinical relevance since EtOH and THC simultaneous use were shown to enhance cognitive impairments [44].

### 3.3. Age Modulates THC- and EtOH-Induced Brain Damage

Several factors can modulate the responses to cannabinoids and explain the variability of clinical symptoms. Exposure characteristics (delivery route, acute or chronic use, frequency), interactions with food (diet, health problems, and medications), and genetic susceptibility factors (THC-metabolizing pathways, cannabinoid receptor genes polymorphism, and epigenetic) can vary significantly between patients [7,55]. Thus, it has been proposed that about 20% of the variance in the vulnerability to drug dependence might be due to common single nucleotide polymorphisms [56] and, for instance, whether human ABCB1 influences cannabis-related phenotypes is under debate [57].

Besides these factors, age, another individual key parameter might modulate the cannabis effects. Indeed, stroke appeared more frequent in young people [20,21,58,59,60]. This is important since even if the prognosis of stroke is relatively favorable in young people, some of them might experience persisting sequelae [61].

Both the vascular and the cellular hypothesis deserve discussions. The temporal relationship between cannabis consumption and stroke occurrence in the young, together with the reversible cerebral vasoconstriction triggered by cannabis involved in one-third of ischemic strokes, support a vascular contribution to stroke in the young. Additionally, Gueyraud et al., found that cannabis use was associated with cerebrovascular atherosclerotic lesions in young adults with ischemic stroke [23]. Accordingly, cerebral vasoconstriction can be observed after natural or synthetic cannabis use [62,63].

In addition, a direct cellular effect of cannabis on brain mitochondria is also likely to be involved in the occurrence of stroke. To the best of our knowledge, this study is the first comparing the mitochondrial effects of THC and alcohol on young and middle-aged brains. The results demonstrate that young age is associated with enhanced deleterious effects of both drugs. These data support that young brains are more sensitive to EtOH and THC, and are in accordance with the greater impairments of spatial learning and memory in adolescents as compared to adult rats.

Indeed, the adolescent period is crucial. The adolescent brain is still immature and likely show increased vulnerability to harmful environmental influences, including THC and EtOH use [64,65,66]. Accordingly, cannabis can significantly impair the maturation of neural circuits and, although controversial data were published depending on the cerebral localization involved, long lasting cognition alterations were reported in rodents chronically exposed to cannabinoids during adolescence (see [10]). Accordingly, an adverse impact on working memory performance was observed in the young, and it appeared that immaturity can be deleterious through reduced protection against damaging factors [67,68,69,70].

## 4. Materials and Methods

### 4.1. Study Design

Human brains being not easily available, although caution should apply when translating experimental data to human beings, rats appeared generally as adequate models to investigate brain functions. Accordingly, a recent work demonstrated similar THC-related increase in risky decision-making in both human and rats exposed to high-doses of THC [70]. The study was therefore performed on young 11-week-old (n = 5) and middle-aged 45-week-old (n = 5) male Wistar rats (Janvier, Le Genest-St-Isle, France). The number of animal per group allows adequate statistical analysis, as previously reported, based on a power calculation of 80% and α risk of 5% (G*power software 3.1.9.7, Heinrich-Heine-University, Düsseldorf, Deutschland), [27,43]. Acknowledging that female rats also need to be studied (see limitations), we firstly investigated male rats since most THC-related strokes occurs in men.

Animals were housed with an enriched environment at 22 ± 2 °C and a 12 h light–dark cycle, water, and food ad libitum. The experiment was performed in accordance with the principles of laboratory animal care and respected the European Union Guidelines (86/609/EU) and the Committee for the Care and Use of Laboratory Animals (Cremeas, France, decree 2020-274, article. R. 214-89).

By placing them in an induction chamber (Minerve, Esternay, France) with 3% isoflurane, the rats were anesthetized and decapitated. Excised brains were immediately placed in an ice-cold isolation buffer (50 mM Tris, 70 mM sucrose, and 210 mM mannitol, pH 7.4 at +4 °C).

We investigated the dose–response effects of ethanol alone and of synthetic THC (diluted 25 mg/mL in ethanol, Sigma Aldrich, St. Louis, MO, USA) on the brain on mitochondrial respiration. Synthetic THC, as compared to natural extracts, is easier to obtain and likely of constant quality. Indeed, to study the THC used in real life by consumers might have been more realistic, but its composition largely varies and it is rarely pure, particularly when it is from an illegal source [71,72]. Further, synthetic cannabinoids also induce deleterious effects. They were firstly synthesized to improve the therapeutic effects of cannabis, but their increasing misuse became a major issue [73]. Thus, very young children accidentally ingest synthetic cannabinoid containing THC and present with adverse effects like lethargy, altered mental status, and increased muscle tone [74].

Ethanol was injected alone with concentrations ranging from 0.11 × 10^−5^, 0.22 × 10^−5^, 0.33 × 10^−5^, 0.44 × 10^−5^, 0.55 × 10^−5,^ and 0.66 × 10^−5^ M. These injections correspond to the concentration needed to dissolve THC. Thus, THC dissolved in ethanol was injected in the respiration chamber at the following concentrations, increased every 4 min: 1 × 10^−5^, 2 × 10^−5^, 3 × 10^−5^, 4 × 10^−5^, and 5 × 10^−5^ 6 × 10^−5^ M, based on previous data [27,28,43]. Indeed, THC needed to be dissolved to be studied in the respiratory chamber. We used EtOH as a solvent since it has one of the lower toxicity as compared to other alcohols [75], and since it is often used concomitantly with THC by humans.

### 4.2. Mitochondrial Extraction

As previously reported [76,77], mitochondria were extracted using sequential centrifugation. After washing, the brain was homogenized with a MACS Dissociator (Miltenyi Biotec, Bergisch Gladbach, Germany). Following three minutes centrifugation at 3000 rpm at 4 °C, the supernatant was centrifuged at 8000 rpm during 10 min at 4 °C. Then, mitochondria were washed and concentrated by centrifugation at 11,000 rpm, during 5 min at 4 °C. The pellet of mitochondria was suspended with an ice-cold buffer (50 mM Tris, 70 mM sucrose, and 210 mM mannitol, pH 7.4 at +4 °C), and protein content was quantified with a Bradford assay.

### 4.3. Mitochondrial Respiration

Brain mitochondrial respiration was determined using a high-resolution oxygraph (Oxygraph-2K, Oroboros instruments, Innsbruck, Austria), containing 2 clark-type electrodes, as previously reported [27]. 0.15 mg mitochondria were placed in 2 mL of respiration buffer (Miro5+creatine: EGTA 0.5 mM, MgCl_2_ 3 mM, K lactobionate 60 mM, Taurine 20 mM, KH_2_PO_4_ 10 mM, HEPES 20 mM, Sucrose 110 mM, BSA 1 mg/mL, Creatine 20 mM). The registration started by the measure of OXPHOS CI after injection of glutamate (10 mM), malate (2.5 mM), and ADP (2 mM), at 37 °C under continuous stirring. Then, we injected six increasing doses of ethanol alone, or EtOH and THC, as described above. We expressed mitochondrial respiration rates as pmol O_2_/s/mL.

### 4.4. Mitochondrial H_2_O_2_ Production

We determined mitochondrial hydrogen peroxide (H_2_O_2_) production using amplex red and Horse Radish peroxidase (HRP) with the O2K-Fluo LED2-Module. When amplex red (20µM) found a molecule of H_2_O_2_, HRP (1 U/mL) metabolizes this couple on a fluorescent molecule, resorufin (563 nm/587 nm) [78]. The mitochondrial substrates added were glutamate, malate, and ADP, in the same concentration as described in the methods for mitochondrial respiration analysis. Data were expressed in pmol/s/mL.

### 4.5. Statistical Analysis

All data were expressed as mean ± standard error of the mean (SEM). The statistical analyses were performed using Prism software (GraphPadPrism 8.4.3, GraphPad Software, San Diego, CA, USA). After checking normality with the Shapiro–Wilk test, one-way ANOVA was performed with the Dunnett post hoc test to analyze the parameters’ evolution following THC or vehicle exposures. We applied Dunn’s test to correct the ANOV, as recommended.

For the samples, following a normality test, a student’s two-tailed t-test was used for group comparisons, and for other comparisons, a Mann–Whitney test was performed. A *p*-value < 0.05 was considered statistically significant.

## 5. Limitation and Perspectives

The study’s small sample size might be a limitation, but power analysis supported this number, allowing for adequate statistical analysis.

As for all animal models, caution should apply when translating experimental data to human beings. Nevertheless, rats are often used in studies investigating brain function, and a recent work demonstrated similar THC-related increase in risky decision-making in both human adolescents and rats exposed to high-dose THC [70]. This suggests that our data might be relevant in human brains, but it needs further confirmation in larger population.

In this study, the control was the baseline mitochondrial respiratory values before drug addition. Continuously and separately measuring brain mitochondrial respiration without adding any drug might have been useful, but the period of the study was relatively brief and a spontaneous and significant impairment in mitochondrial respiration occurrence seems unlikely. Indeed, nanoparticles addition failed to modify brain mitochondrial respiration, suggesting its relative stability over the time-period of the present study [77].

Among young subjects, alcohol is the first drug used, and THC is usually taken later. Since Et-OH affects the brain by itself, subsequent treatment of the brain with a combination of both drugs may induce more severe changes than when the brain first encounters both substances at once. Thus, further studies investigating the effect of combined EtOH and THC on brain mitochondria will be interesting to perform in rats already taking EtOH for several weeks.

Finally, the study focuses exclusively on male rats, which limits its generalizability. Including female rats would provide a more comprehensive understanding of potential sex-based differences in mitochondrial function and substance response. Indeed, gender is also a factor modulating the toxicity of THC. Thus, sex modifies THC pharmacokinetics, with mean values of THC being greater in women than in men [79]. Accordingly in rats, sub chronic THC impaired rats undergo spatial learning differently depending on sex [64]. Also, the development of psychopathology appeared sex-differentiated after cannabis exposure during adolescence [67]. Similarly, the effects of acute EtOH show sex differences [80].

## 6. Conclusions

In conclusion, these data support the idea that subcellular alterations, at the mitochondrial level, participate in acute brain damage-related to cannabis and EtOH use. Importantly, both young age and concomitant drug use enhanced mitochondrial respiration impairments. Thus, increased sensitivity of young brain mitochondria might be involved in the higher occurrence of cannabis-related stroke in young people. Further studies will be useful to address the impact of chronic exposures and the role of other predisposing factors (gender, genetic, and epigenetic).

Given the prevalence of alcohol and cannabis co-use among college students and their higher risk as compared to adult consumers, increased prevention strategies against THC and EtOH co-use are crucial [23,81,82]. Although challenging, cooperation between consumers, parents, and service providers should allow a reduction in cannabis-related harm, and should decrease impaired neurodevelopmental processes and network efficiency, which is associated with altered white and gray matter, dendritic structure, and synaptic functions. Indeed, even short-term abstinence might reduce verbal learning and working impairments in adolescent [83,84,85,86].

## Data Availability

The original contributions presented in this study are included in the article. Further inquiries can be directed to the corresponding author.
